# Supplementary material for: The signaling axis atypical protein kinase C λ/ι-Satb2 mediates leukemic transformation of B-cell progenitors
Source: Nat Commun. 2019 Jan 4;10:46. doi: 10.1038/s41467-018-07846-y (PMC6320370; doi:10.1038/s41467-018-07846-y)

SUPPLEMENTARY FIGURES

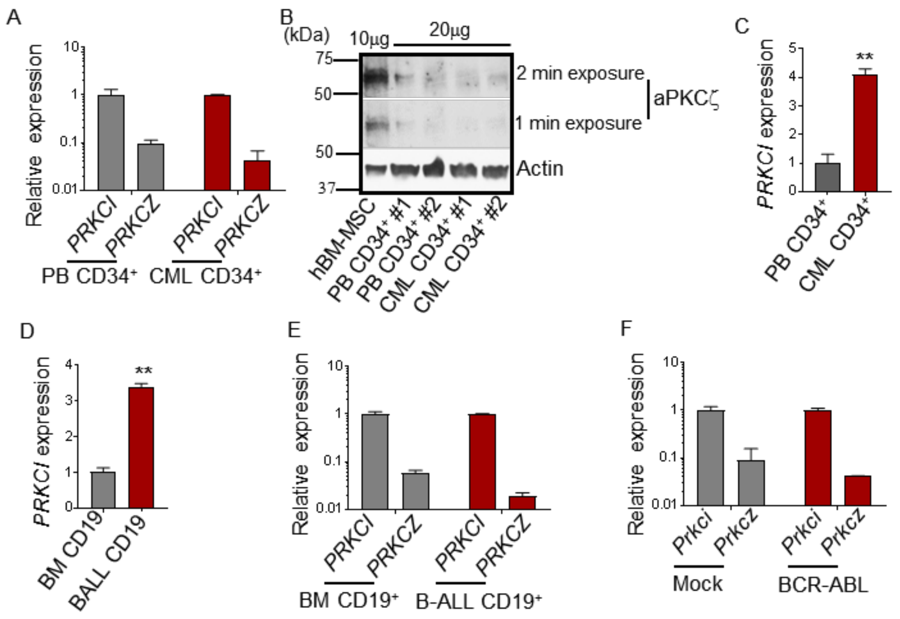

Supplementary Figure 1. Atypical protein kinase Cλ is predominantly expressed in normal and leukemic stem and progenitors. A) Quantitative real time PCR (Q-RT-PCR) analyses of *PRKCZ* and *PRKCI* in healthy donor and chronic myelogenous leukemia patients' derived $CD34^+$ cells. B) Western blot analyses of aPKCζ in $CD34^+$ cells from healthy donors and chronic myelogenous leukemia patients. Human bone marrow mesenchymal stem cells (h-BM MSC) was taken as a positive control for the expression of aPKCζ. For $CD34^+$ cells, higher amount (20 μg) of protein was loaded into each lane to detect minimal level of aPKCζ expression. C) Q-RT-PCR analyses of *PRKCI* in CD34+ cells from healthy donor (n=2) and CML patients (n=2). D) Q-RT-PCR analyses of *PRKCI* in B-cell progenitors ($CD34^+$ $CD19^+$) from healthy donor (n=2) and B-ALL patients (n=2). E) Quantitative real time PCR (Q-RT-PCR) analyses of *PRKCZ* and *PRKCI* in B-cell progenitors from healthy donor (n=2) and B-ALL patients (n=2). F) Q-RT-PCR analyses of *Prkcz* and *Prkci* in normal and BCR-ABL transduced murine hematopoietic stem and progenitors. Western blot images of whole blots are presented as a separate supplementary file. Data are presented as mean ± SD of a minimum of 2 independent experiments. $^{**}p<0.01$, t-test.

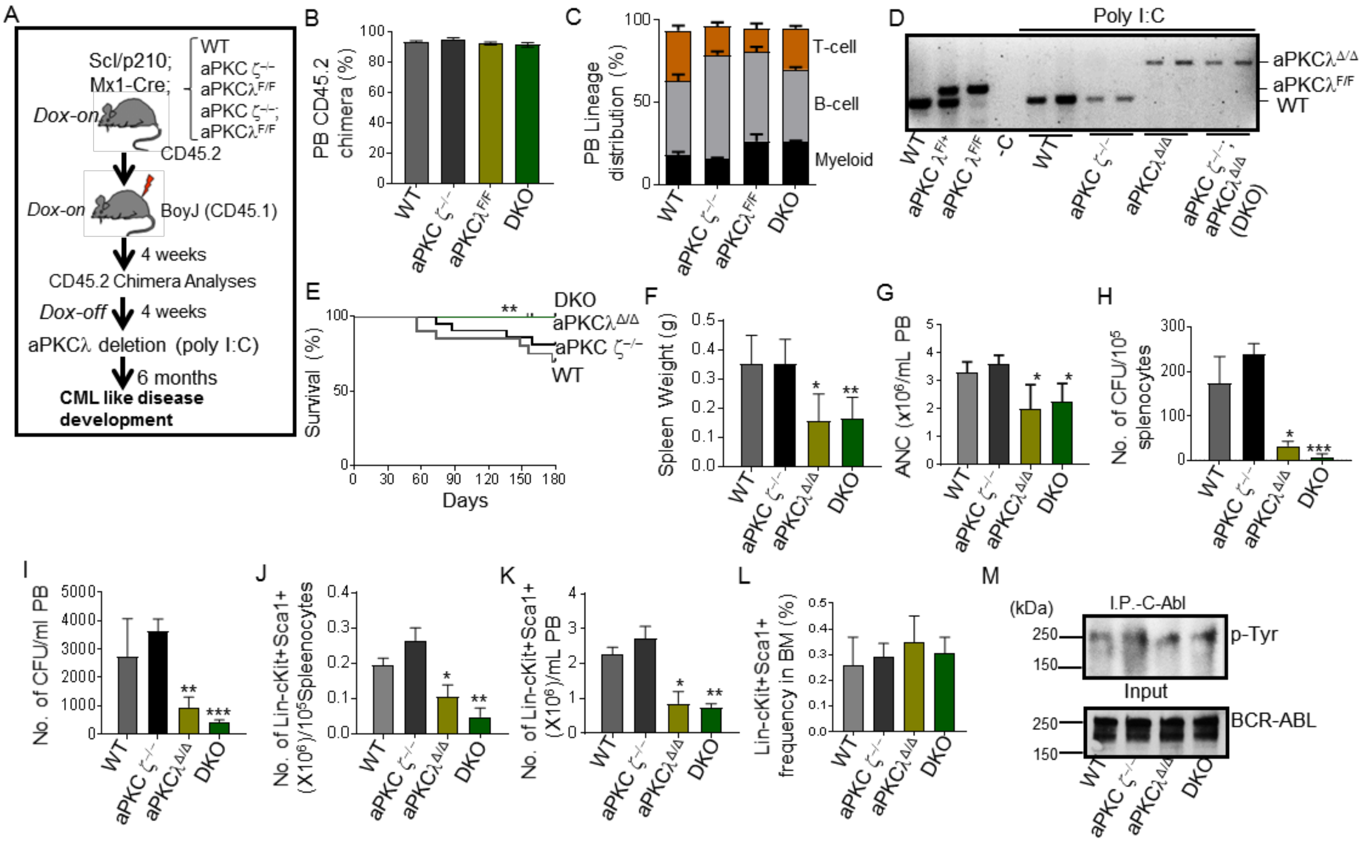

Supplementary Figure 2. Atypical protein kinase Cλ is required for the development of p210-BCR-ABL induced chronic myelogenous leukemia. A) Schematic diagram to investigate the role of aPKCζ and aPKCλ in the development and progression of p210-BCR-ABL induced CML-like disease. Whole BM cells from *Dox-Off* inducible (Scl/p210) WT, aPKC$\zeta^{-/-}$, aPKC$\lambda^{F/F}$ and aPKC$\zeta^{-/-}$; aPKC$\lambda^{F/F}$ group were transplanted into B6.SJL.Ptprc^{a}.Pepc$^{b\ BoyJ}$ (Boy J) primary recipient mice. Expression of p210-BCR-ABL oncogene was induced by doxycycline withdrawal (*Dox-off*) from the diet, and aPKCλ was deleted by poly I:C administration. Mice were followed for CML-like disease development. B) FACS quantification of CD45.2^{+} chimera in the PB of primary chimeric mice 4 weeks post transplantation before the induction of p210-BCR-ABL expression. C) FACS quantification of the lineage distributions in the PB of primary recipients 4 weeks post initiation of the induction of p210-BCR-ABL (before poly I:C administration for aPKCλ deletion). Solid bars: myeloid. Grey bars: B-lymphoid. Orange bars: T-lymphoid. D) Representative examples of polymerase chain reaction (PCR) amplification of un-recombined mouse tails (3 lanes on the left side) and blood DNA specimens (8 right lanes) showing inducible deletion of the floxed alleles of aPKCλ through Poly I:C administration. –C: control without DNA. aPKC$\lambda^{F/F}$ chimeric mice are designated as aPKC$\lambda^{\Delta/\Delta}$ after polyI:C induced floxed alleles, and aPKC$\zeta^{-/-}$;aPKC$\lambda^{F/F}$

as DKO. E) Kaplan-Meier survival analyses of primary recipient mice. 20% of the mice in WT and aPKC$\zeta^{-/-}$ group were died of CML-like disease, and rest of the mice were sacrificed and analyzed 6 months post poly I:C administration. F) Spleen size at autopsy showing splenomegaly in WT and aPKC$\zeta^{-/-}$ group, but not in aPKC$\lambda^{\Delta/\Delta}$ and DKO group. G) PB Absolute neutrophil count showing neutrophilia in WT and aPKC$\zeta^{-/-}$ transplanted mice. H-I) CFU-C content in the splenocytes (H) and peripheral blood (I) of the leukemic mice. J-K) FACS quantification of leukemic stem cells and progenitors (CD45.2^{+} Lin^{-} cKit^{+} Sca1^{+}) in the spleen (J) and peripheral blood (K) of primary recipient leukemic mice. L) Frequency of LSK (%) in the BM of WT, aPKC$\zeta^{-/-}$, aPKC$\lambda^{\Delta/\Delta}$ and DKO chimeric mice. M) Expression and activation of p210-BCR-ABL in hematopoietic stem and progenitor cells derived from WT, aPKC$\zeta^{-/-}$, aPKC$\lambda^{\Delta/\Delta}$ and DKO leukemic chimeric mice. Data are presented as mean ± SD of a minimum of three independent experiments. *p<0.05; **p<0.01, t-test.

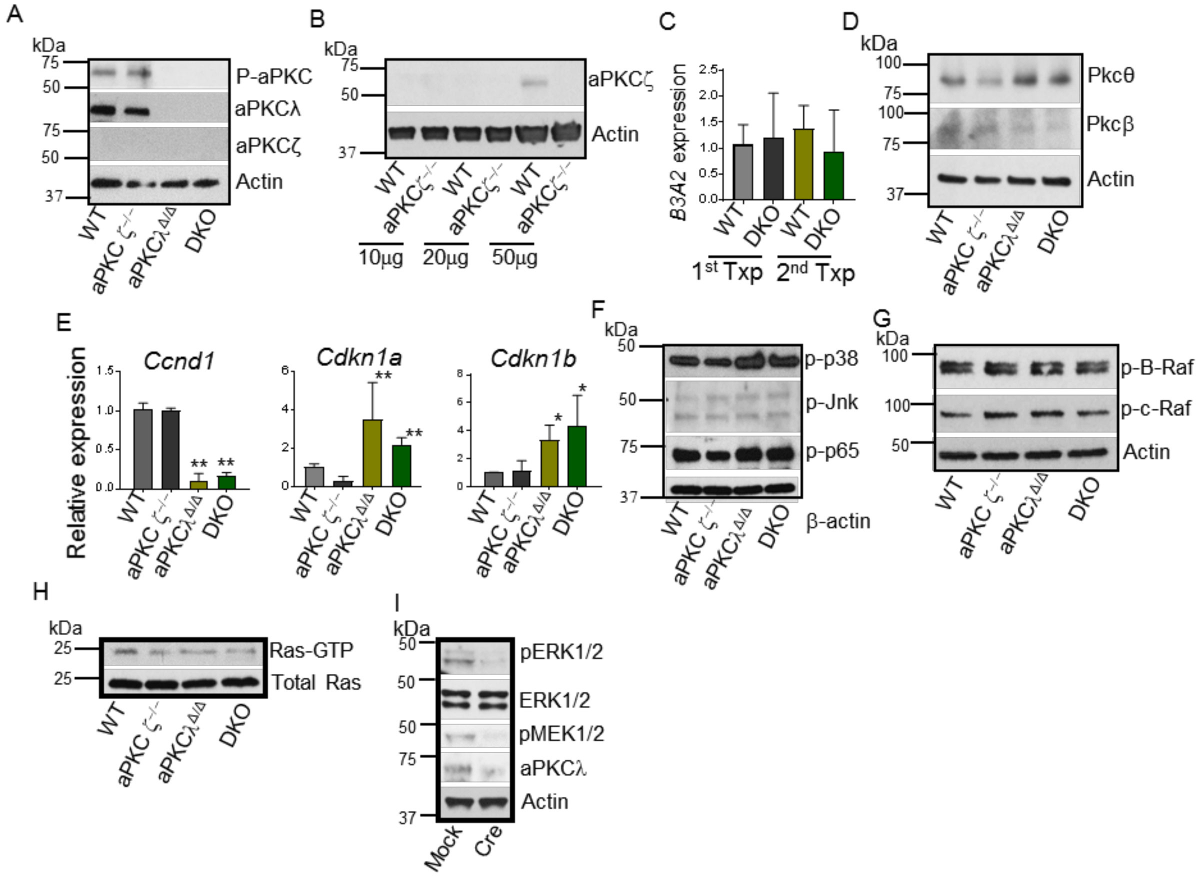

Supplementary Figure 3. Deficiency of aPKCλ or both aPKCλ and -ζ lead to impaired cell cycle progression and attenuated activation of Rac GTPases. A) Representative western blot analyses of phospho-aPKC, aPKCζ, aPKCλ and Actin in leukemic progenitors from leukemic murine BM. B) Western blot analyses of aPKCζ in leukemic B-cell progenitors. Expression of aPKCζ is detectable in lane loaded with 50 µg of whole cell lysate. C) Q-RT-PCR analyses of p210-BCR-ABL (B3A2) expression in $Lin^{-}cKit^{+}$ (LK) cells derived from primary and secondary recipient chimeric mice. D) Representative western blot analyses of PKCβ, PKCθ and Actin in WT and aPKC deficient leukemic B-cell progenitors. E) Q-RT PCR analyses showing relative expression of *Ccnd1, Cdkn1a, Cdkn1b* in WT, aPKC$\zeta^{-/-}$, aPKC$\lambda^{\Delta/\Delta}$ and DKO leukemic B-cell progenitors. F) Representative western blots of p-P38, p-JNK, p-p65, aPKCλ and Actin in WT, aPKC$\zeta^{-/-}$, aPKC$\lambda^{\Delta/\Delta}$ and DKO leukemic B-cell progenitors. G) Representative example of western blots of phospho-c-Raf, phospho-B-Raf , and Actin in WT, aPKC$\zeta^{-/-}$, aPKC$\lambda^{\Delta/\Delta}$ and DKO leukemic B-cell progenitors. H) Representative example of the analyses of Ras GTPase activation by specific effector pulldown assay in leukemic B-cell progenitors derived from WT, aPKC$\zeta^{-/-}$, aPKC$\lambda^{\Delta/\Delta}$ and DKO chimeric mice. I) Representative western blots of phospho-ERK1/2, ERK1/2, phspho-MEK1/2, aPKCλ and Actin in BCR-ABL+ hematopoietic stem and progenitors transduced with

empty vector or Cre recombinase expressing lentiviruses. Western blots are representative of a minimum of 2 or more independent experiments. Q-RT-PCR data are presented as mean ± SD of three independent experiments. *$p<0.05$; **$p<0.01$, t-test.

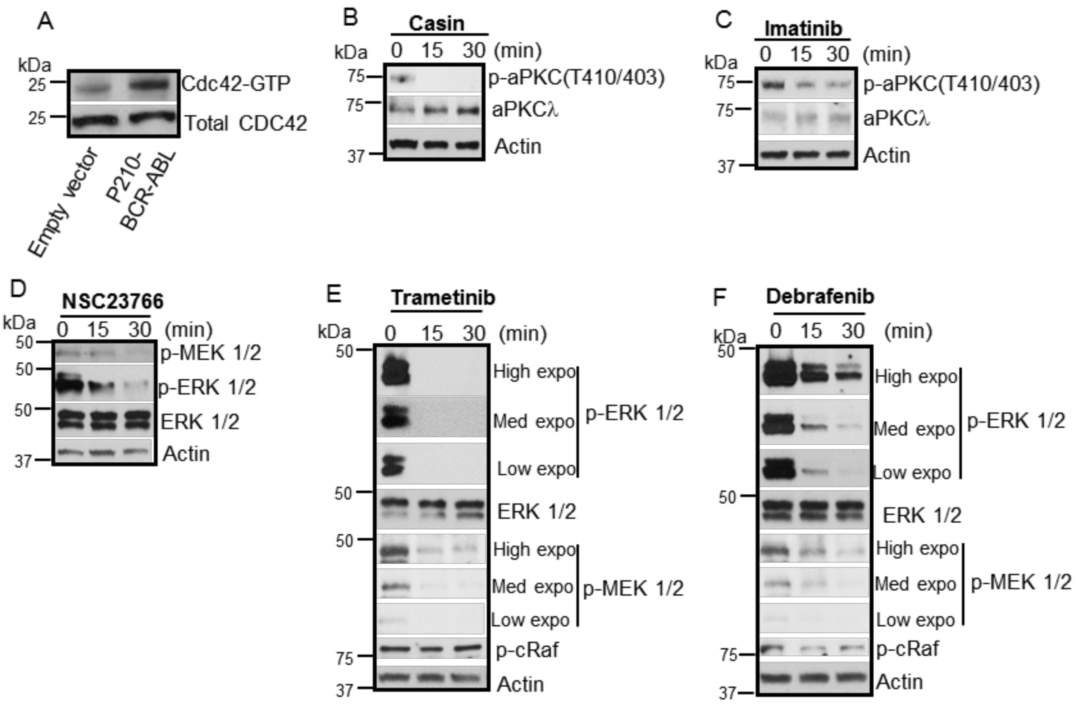

Supplementary Figure 4. Atypical PKCλ downstream of BCR-ABL and Cdc42 controls activation of Rac GTPase, MEK-ERK MAPK pathway. A) Representative example of the analyses of Cdc42 GTPase activation by specific effector pulldown (PAK-PBD agarose) assay in leukemic stem and progenitors derived from WT chimeric mice. B-C) Representative western blot analyses of phospho-aPKC, aPKCλ and Actin in Casin (B) and Imatinib (C) treated B-cell progenitors. Cdc42 inhibition completely abrogates aPKCλ phosphorylation. Imatinib downregulates aPKCλ activation. D) Representative western blot analyses of phospho-Mek1/2, phospho-Erk1/2, ERK1/2 and actin in leukemic B-cell precursors treated with NSC23766 for different time. E-F) Western blot analyses phospho-Erk1/2, Erk1/2, phospho-Mek1/2, phospho-C-Raf and Actin in WT leukemic B-cell progenitors treated with MEK inhibitor Trametinib (E) and Debrafenib (F). Western blots with different exposure time for phospho-Erk1/2 and phospho-Mek1/2 were presented; Low (15 sec), med (30 sec), high (1 min). Experiments were performed using pools of primary leukemic B-cell progenitors from 3 mice for each group and experiment, in two independent experiments.

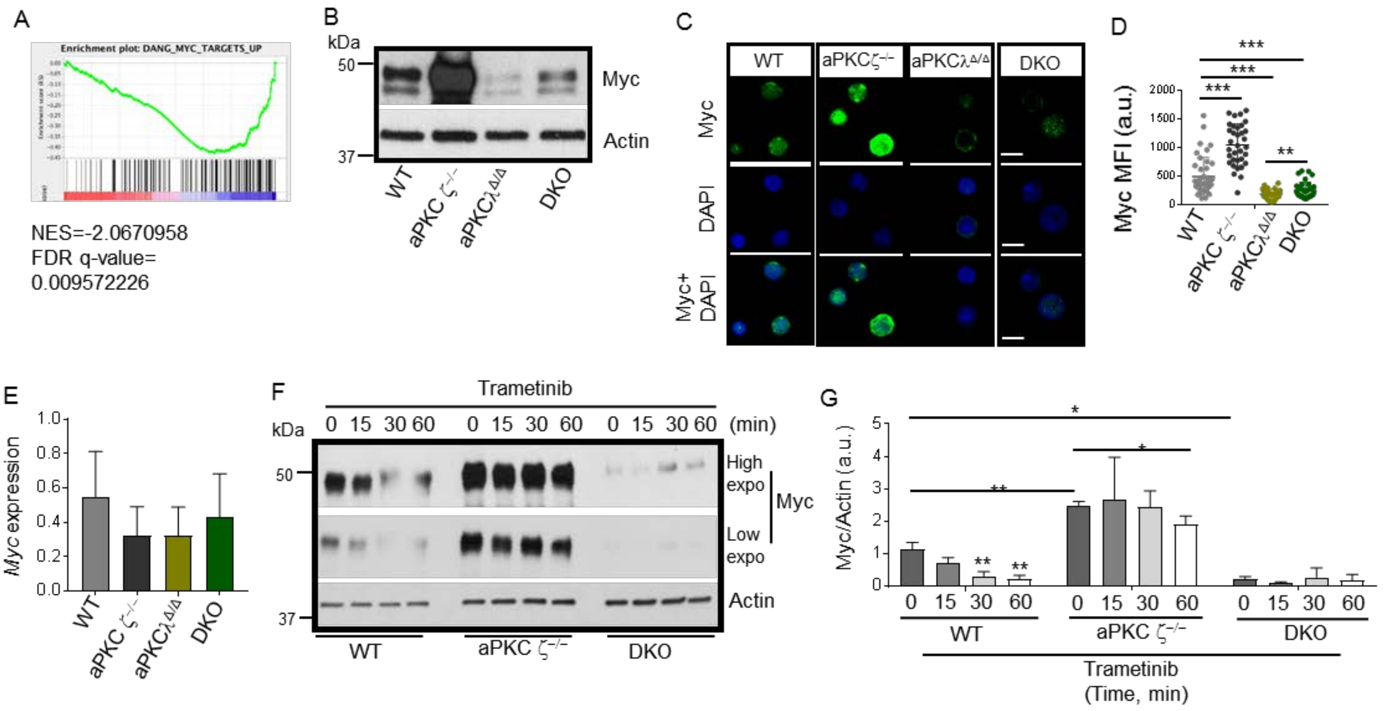

Supplementary Figure 5. Atypical PKCλ and –ζ antagonistically regulate Myc expression. A) Gene set enrichment analyses of whole transcriptome of WT and DKO leukemic B-cell precursors. B) Representative western blots of Myc and Actin in WT, aPKCζ$^{-/-}$, aPKCλ$^{\Delta/\Delta}$ and DKO leukemic B progenitors. C) Confocal microscopic images of the expression and distribution of Myc in WT, aPKCζ$^{-/-}$, aPKCλ$^{\Delta/\Delta}$ and DKO leukemic B progenitors. Bar=10 μm. D) Quantification of mean fluorescence intensity of Myc expression. E) Q-RT-PCR analyses of *Myc* expression in WT, aPKCζ$^{-/-}$, aPKCλ$^{\Delta/\Delta}$ and DKO leukemic B progenitors. F) Western blot analysis of Myc expression in WT, aPKCζ$^{-/-}$ and DKO leukemic B progenitors treated with 20nM Trametinib to inhibit Mek-Erk activation. Myc blots with lower (15 sec) and higher (1 min) exposure were presented to show Myc expression in DKO cells. G) Quantification of western blot band intensity of Myc normalized to Actin in Trametinib treated B-cell progenitors. Experiments were performed using pools of primary leukemic B-cell progenitors from 3 mice for each group in two independent experiments. Mean fluorescence intensity and Myc band intensity were presented as mean ± SD of two independent experiments. *$p<0.05$; **$p<0.01$, t-test.

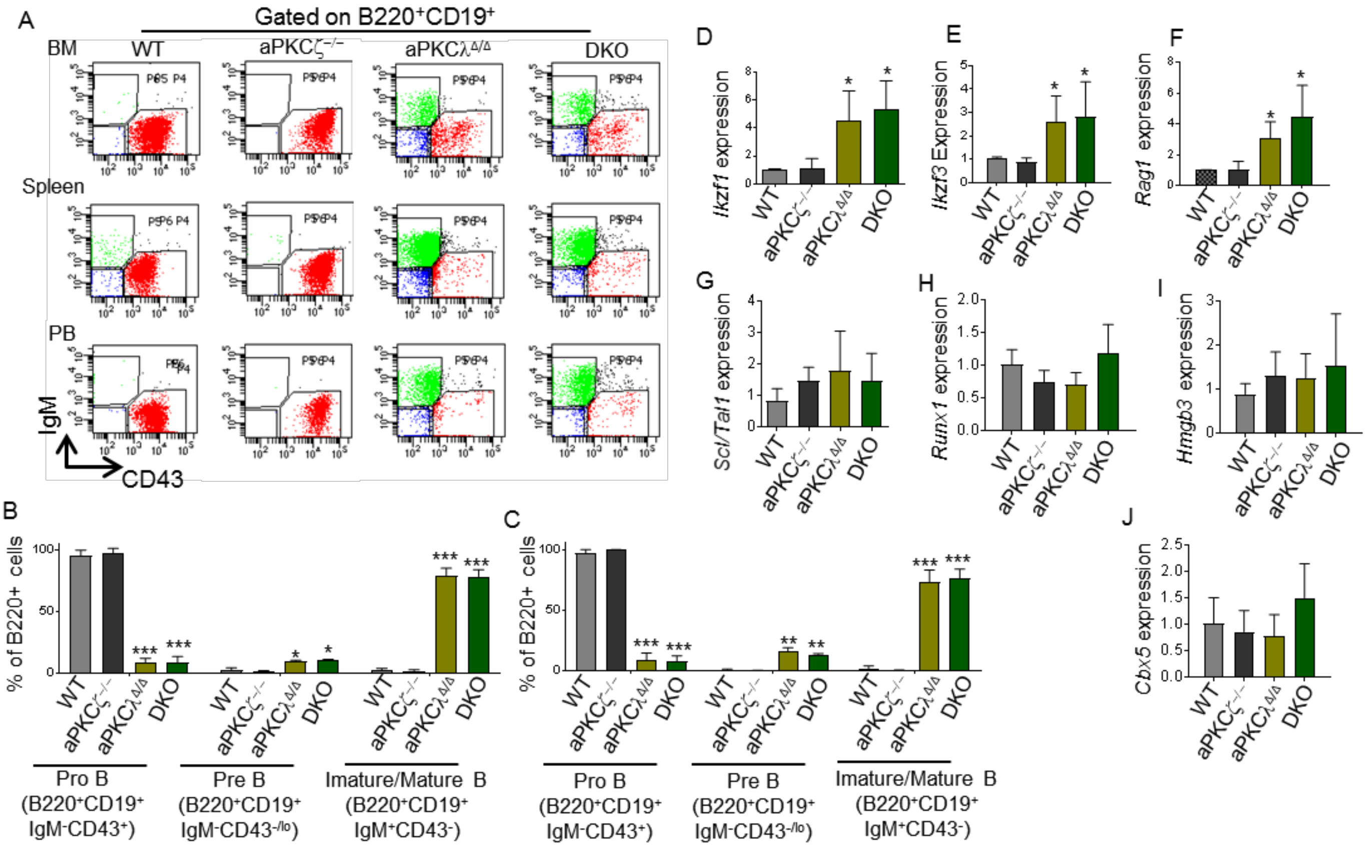

Supplementary Figure 6. Inducible deletion of aPKCλ impaired the differentiation arrest of leukemic B cell progenitors. A) Representative FACS dot plots showing B-cell progenitors and mature/immature B cells in the BM, Spleen and PB of WT, aPKCζ$^{-/-}$, aPKCλ$^{\Delta/\Delta}$ and DKO chimeric secondary recipients. B-C) FACS quantification of leukemic B-cell progenitors and mature/immature B cells in the spleen (B) and PB (C) of WT, aPKCζ$^{-/-}$, aPKCλ$^{\Delta/\Delta}$ and DKO chimeric secondary recipients. D-F) Q-RT-PCR analyses of the expression of *Ikzf1* (D), *Ikzf3* (E) and *Rag1* (F) in WT, aPKCζ$^{-/-}$, aPKCλ$^{\Delta/\Delta}$ and DKO B-cell progenitors. G-J) Q-RT-PCR analyses of the expression of *Scl/Tal1* (G), *Runx1* (H) and *Hmgb3* (I), *Cbx5* (J) in WT, aPKCζ$^{-/-}$, aPKCλ$^{\Delta/\Delta}$ and DKO B-cell progenitors. Data are presented as mean ± SD of three independent experiments. *p<0.05; **p<0.01; ***p<0.001, t-test.

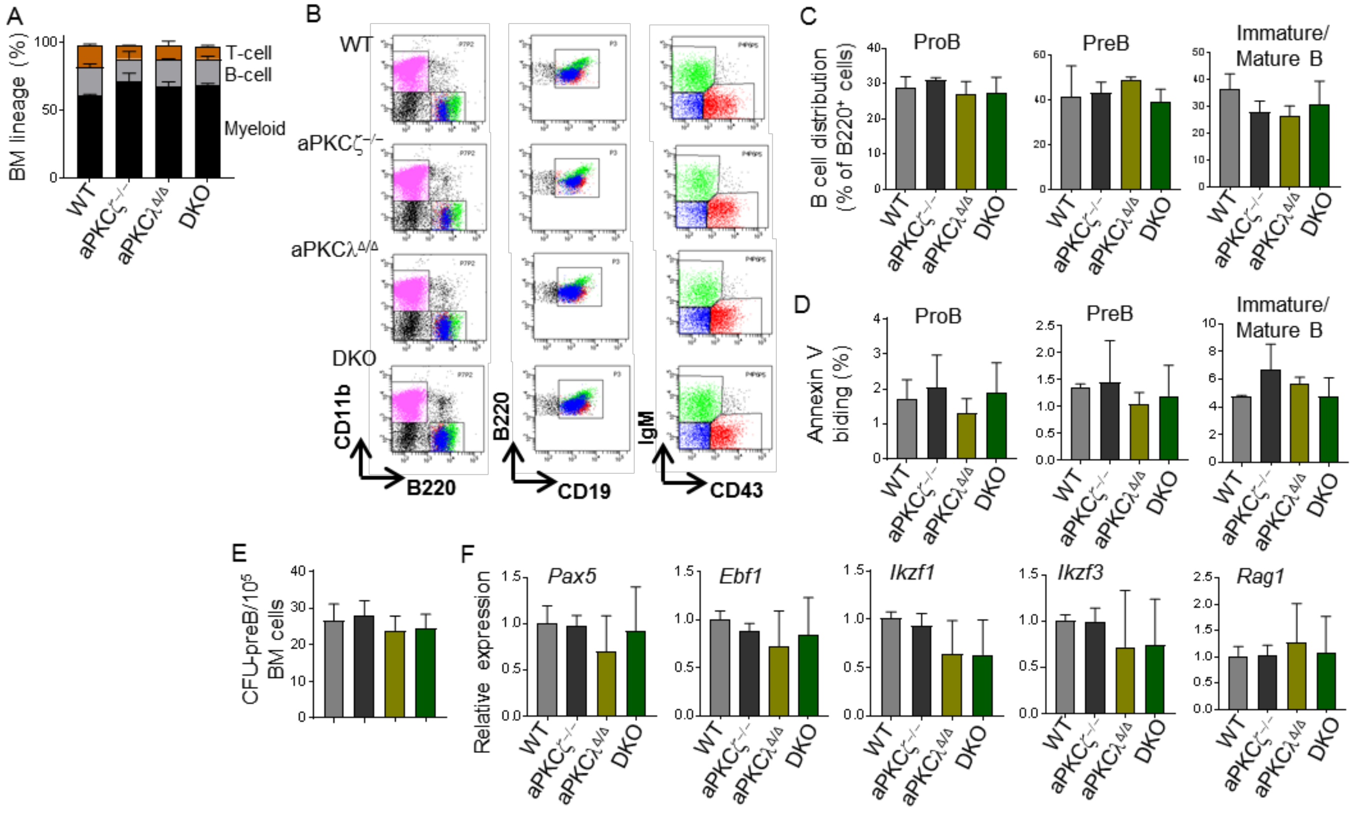

Supplementary Figure 7. Constitutive deletion of aPKCζ and/or Inducible deletion of aPKCλ does not affect normal B-lymphopoiesis in the absence of oncogene expression. A) FACS quantification of BM lineage distribution of Mx1-Cre; WT, aPKC$\zeta^{-/-}$, aPKC$\lambda^{\Delta/\Delta}$ and DKO mice. Solid bars: myeloid. Grey bars: B-lymphoid. Orange bars: T-lymphoid. B) Representative examples of FACS dot plots showing B-cell progenitors population ((Pre-ProB, Pro-B, Pre-B) in Mx1-Cre; WT, aPKC$\zeta^{-/-}$, aPKC$\lambda^{\Delta/\Delta}$ and DKO mice. C) FACS-quantification of Pre-ProB, ProB, PreB and immature/mature B cells in the BM of mice belonging to WT, aPKC$\zeta^{-/-}$, aPKC$\lambda^{\Delta/\Delta}$ and DKO group. D) FACS quantification of the percentage of annexin V binding of WT, aPKC$\zeta^{-/-}$, aPKC$\lambda^{\Delta/\Delta}$ and DKO B-cell progenitors and immature/mature B cells. E) CFU-PreB content of the BM of Mx1-Cre; WT, aPKC$\zeta^{-/-}$, aPKC$\lambda^{\Delta/\Delta}$ and DKO mice. F) Q-RT-PCR Analyses of genes involved in B cell differentiation program (*Pax5, Ebf1, Ikzf1, Ikzf3*, and *Rag1*) in normal BCR-ABL^{-} WT and DKO B-cell progenitors. Data are presented as mean ± SD of three independent experiments.

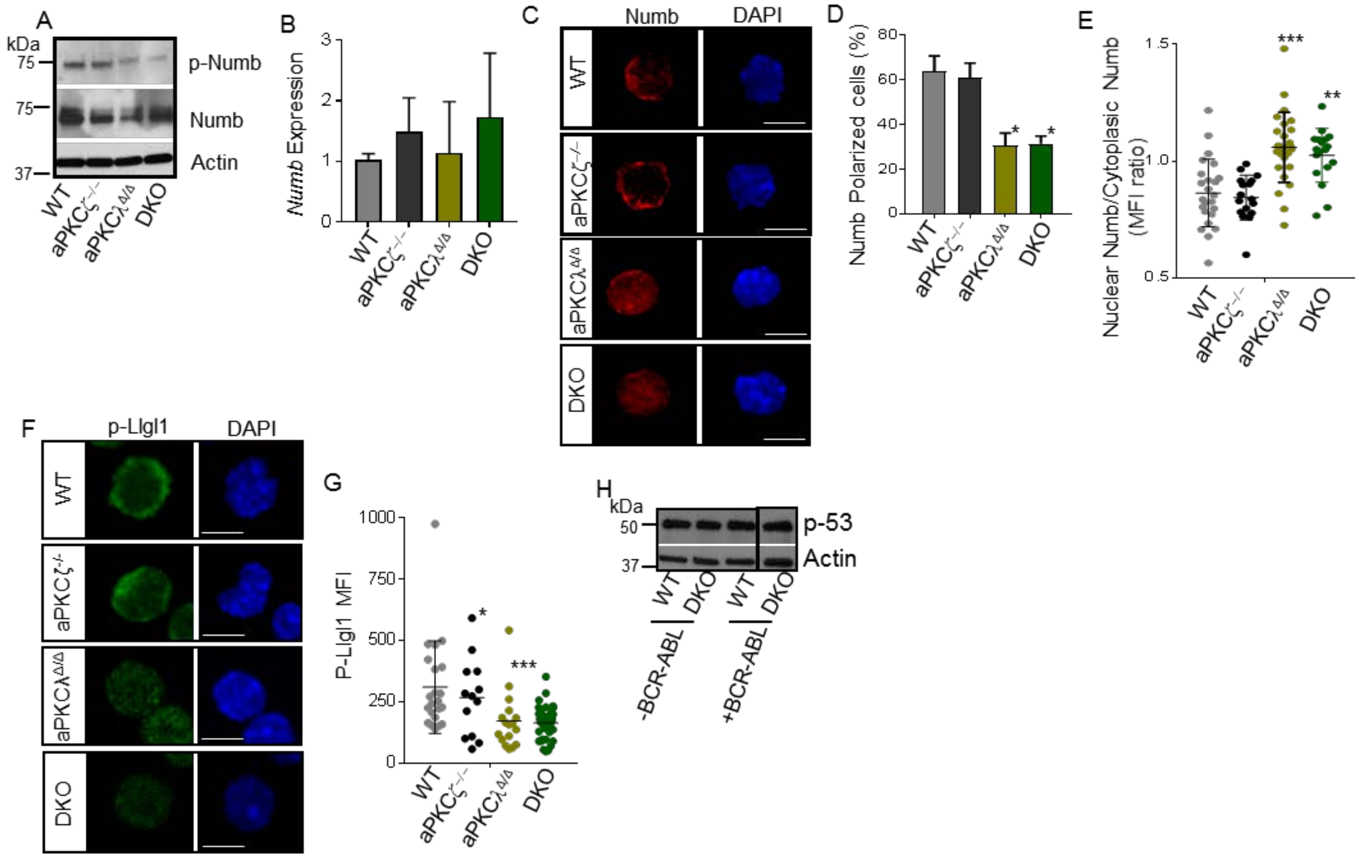

Supplementary Figure 8. Atypical PKCλ deficiency impairs Numb polarization and activation, Lgl1 activation and induces Numb translocation to the nucleus but does not result in increased stabilization of p53. A) Representative example of western-blot of expression and phosphorylation of Numb. B) Q-RT PCR analyses showing relative expression of *Numb* mRNA in WT, aPKC$\zeta^{-/-}$, aPKC$\lambda^{\Delta/\Delta}$ and DKO B-cell precursors. C) Representative example of confocal microscopic images of the distribution of Numb in leukemic B-cell precursors derived from WT, aPKC$\zeta^{-/-}$, aPKC$\lambda^{\Delta/\Delta}$ and DKO mice. Deficiency of aPKCλ impaired polarized Numb distribution leading to nuclear translocation. D) Quantification of the B-cell precursors with asymmetric distribution of Numb. E) Confocal microscopic quantification of the ratio of the nuclear numb to cytoplasmic numb in leukemic B-cell precursors. F) Representative example of confocal microscopic images of the expression and distribution of phospho-Llgl1 in WT, aPKC$\zeta^{-/-}$, aPKC$\lambda^{\Delta/\Delta}$ and DKO B-cell precursors. G) Quantification of mean fluorescence intensity (MFI) of p-Llgl1 in leukemic B-cell precursors. H) Representative example of western blot analyses of p-53 expression in WT/aPKC deficient normal and leukemic B cell precursors. Western blots and confocal images are representative of 2 independent experiments. Data depict mean ± SD. Bar=10μ. *$p<0.05$; **$p<0.01$; ***$p<0.001$, t-test.

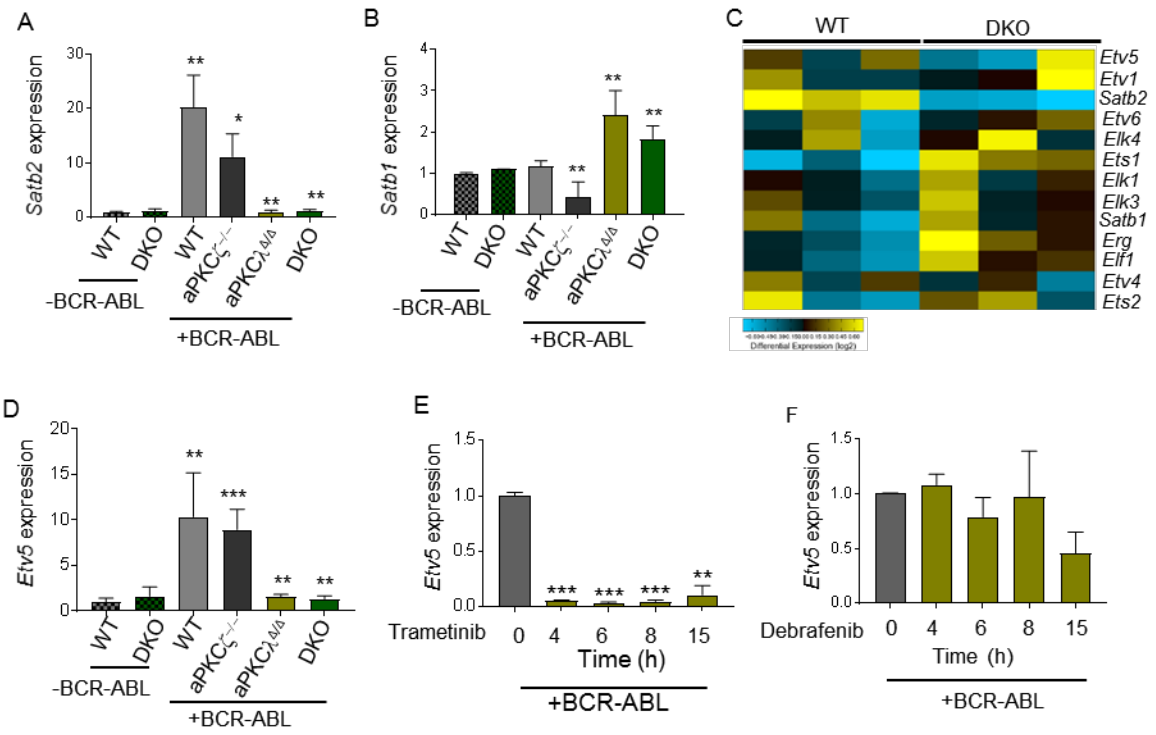

Supplementary Figure 9. Atypical PKCλ mediated Mek1/2-Erk1/2 activation controls Etv5 expression in lymphoid leukemia B-cell progenitors. A-B) Q-RT-PCR analyses of chromatin modifier genes *Satb2* (A) and *Satb1* (B) in normal BCR-ABL^{-} WT and aPKC deficient B cell precursors, and leukemic B-cell precursors derived from WT, aPKCζ$^{-/-}$, aPKCλ$^{\Delta/\Delta}$ and DKO chimeric mice. C) Heat map of differential expression of Ets family transcription factors and chromatin modifiers Satb2 and Stab1 in WT and DKO leukemic B progenitors. D) Q-RT-PCR analyses of *Etv5* in normal BCR-ABL^{-} WT and aPKC deficient B cell precursors, and leukemic B-cell precursors derived from WT, aPKCζ$^{-/-}$, aPKCλ$^{\Delta/\Delta}$ and DKO chimeric mice. E-F) Q-RT-PCR analyses for *Etv5* expression in Trametinib (E) and Debrafenib (F) treated WT leukemic B-cell progenitors. Experiments were performed using pools of leukemic B-cell progenitors from 3 mice for each group, in two independent experiments. Data are presented as mean ± SD of three independent experiments. *p<0.05; **p<0.01, ***p<0.001, t-test.

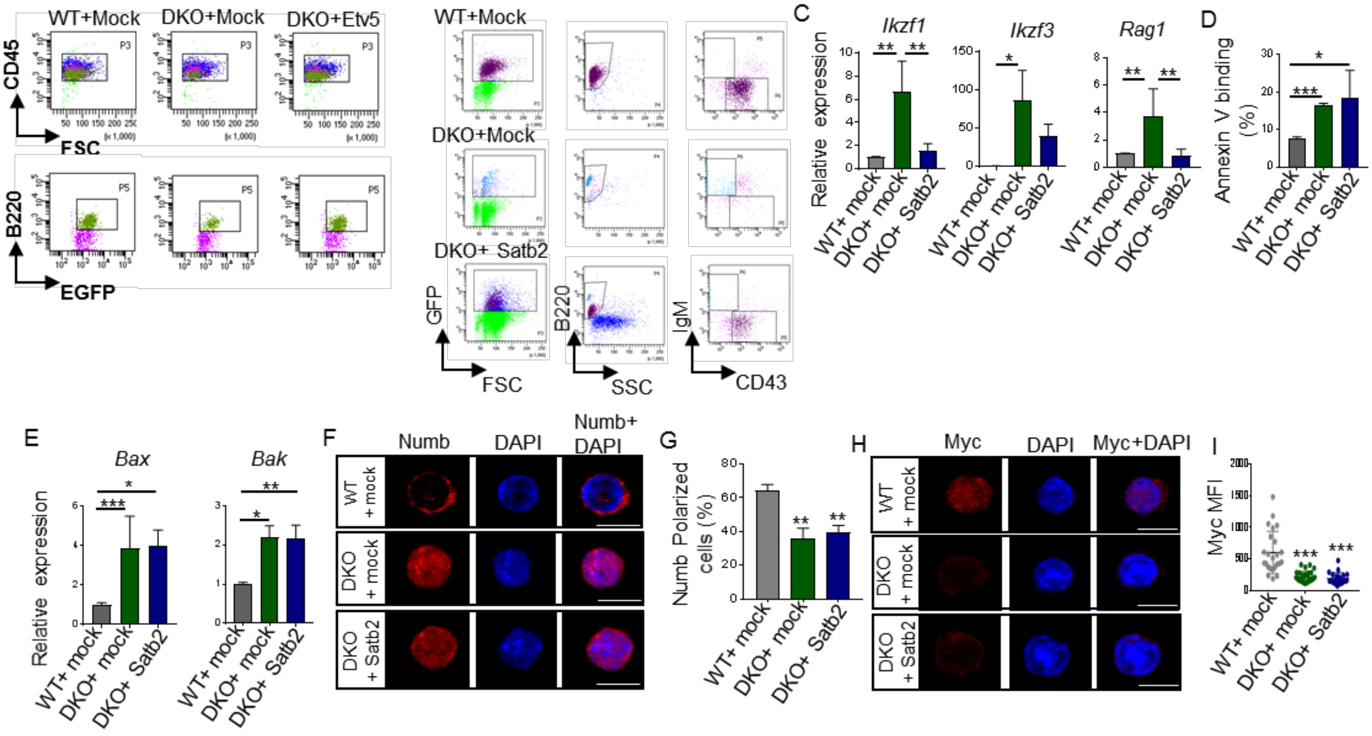

Supplementary Figure 10. Etv5 and Satb2 ectopic expression rescues B cell differentiation arrest of aPKC deficient leukemic B-cell progenitors. A) FACS dot plots showing EGFP+/B220$^{+/}$CD43^{+} B-cell precursors derived from mock/Etv5 transduced LSK cell co-cultured on OP9 stromal monolayer. B) FACS dot plots showing leukemic B-cell precursors in the bone marrow of secondary recipient mice transplanted with mock or Satb2 transduced LSK cells. C) Q-RT PCR analyses of B-cell differentiation genes (*Ikzf1, Ikzf3 and Rag1*) in mock or Satb2 expressing B cell precursors derived from secondary recipients. D) FACS quantification of annexin V binding. E) Q-RT-PCR analyses of the relative expression of BH3-only domain containing pro-apoptotic genes *Bax* and *Bak*. F) Representative example of confocal immunofluorescence microscopic analyses of Numb distribution in mock/satb2 expressing leukemic B cell precursors. Satb2 forced expression does not alter the nuclear Numb distribution in aPKC deficient leukemic B-cell precursors. G) Quantification of the Numb polarized cells. H) Representative confocal images of Myc expression and distribution in mock/Satb2 expressing leukemic B cell precursors. I) Quantification of Myc mean fluorescence intensity (MFI). Bar=10μ. Data are presented as mean ± SD of three independent experiments. Confocal images are representative of 2 independent experiments *p<0.05; **p<0.01, ***p<0.001, t-test.

PKC family genes	DKO	aPKC$\lambda^{\Delta/\Delta}$	aPKC$\zeta^{-/-}$
Prkca	-1.13 (p=0.68)	-1.2 (p=0.51)	-1.09 (p=0.66)
Prkcb	**+4.2 (p=0.0004)**	**+3.2 (p=0.005)**	**+1.8 (p=0.02)**
Prkcd	+1.56 (p=0.13)	+1.7 (p=0.14)	-1.2 (p=0.38)
Prkce	+1.3 (p=0.4)	+1.2 (p=0.63)	-1.09 (p=0.84)
Prkcg	+1.3 (p=0.17)	+1.7 (p=0.06)	**+1.9 (p=0.01)**
Prkch	+3.5 (p=0.12)	+1.7 (p=0.51)	+2.4 (p=0.22)
Prkcq	**+2.9 (p=0.003)**	**+2.27 (p=0.02)**	+1.2 (p=0.2)

Supplementary Table 1. Differential mRNA expression (RNA-seq) of the other non-aPKC members of the PKC family compared with WT B-cell precursors. Numbers depict average differential expression (up-regulation: +; down-regulation: -) and statistical significance of the differences (p). In bold, statistically significant differences ($p<0.05$) are depicted. All statistically significant differences resulted in transcription upregulation which did not explain the genotype associated functional effects through either a consistent oncogenic or tumor suppressor effect of the upregulated Prkcb and Prkcq. Mutant leukemic specimens were compared with WT specimens (n=3 per group; Anova test with Bonferroni correction).

Uncropped images of western blots

Fig1A

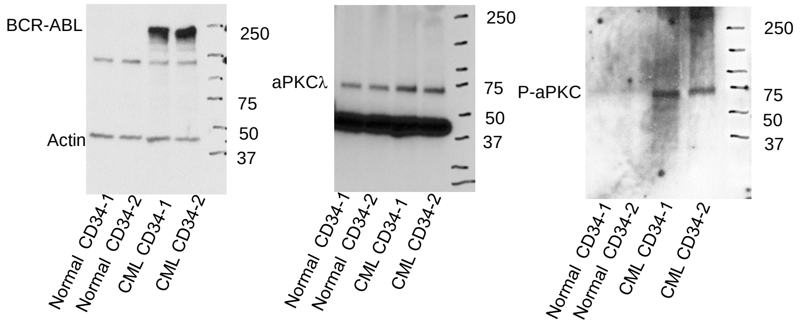

Fig1E

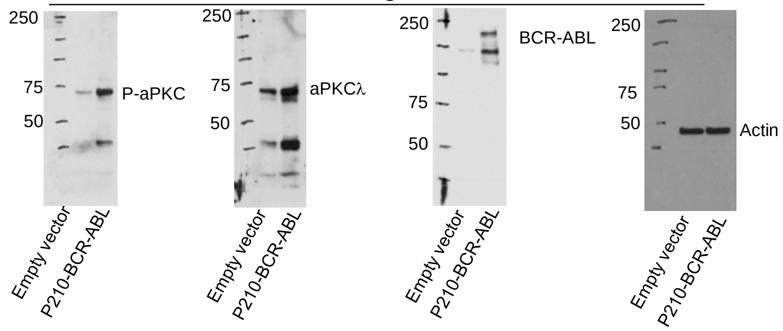

Fig1G

IP

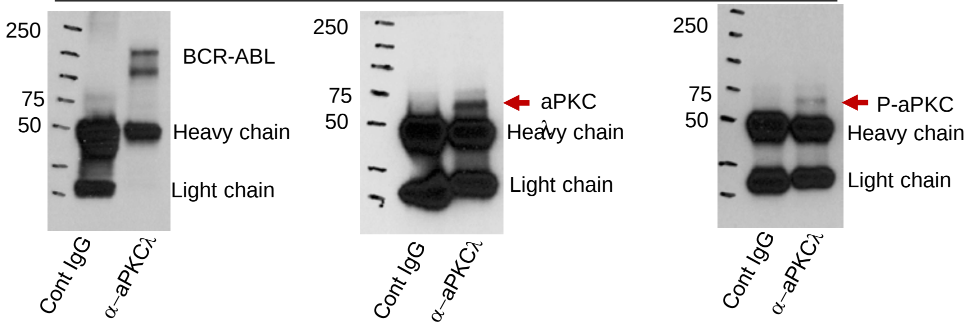

Input

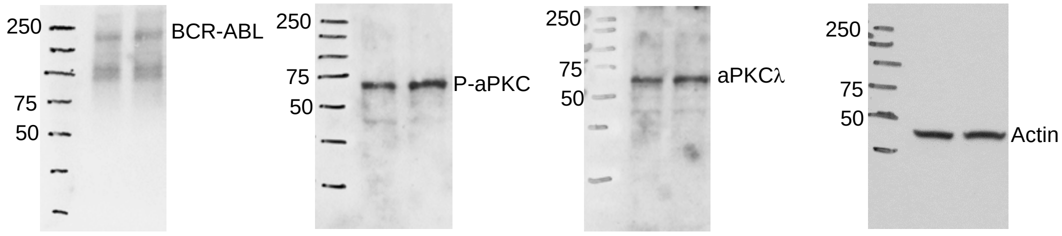

Fig2K

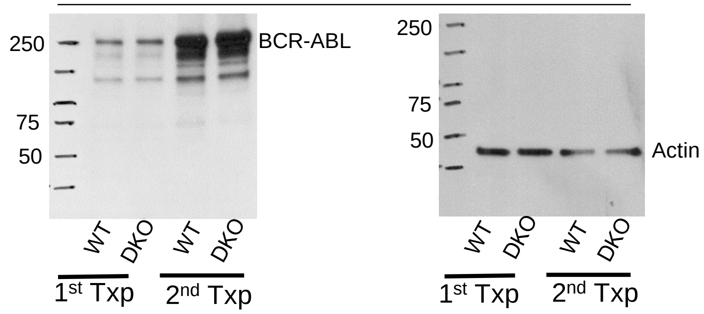

Fig3D

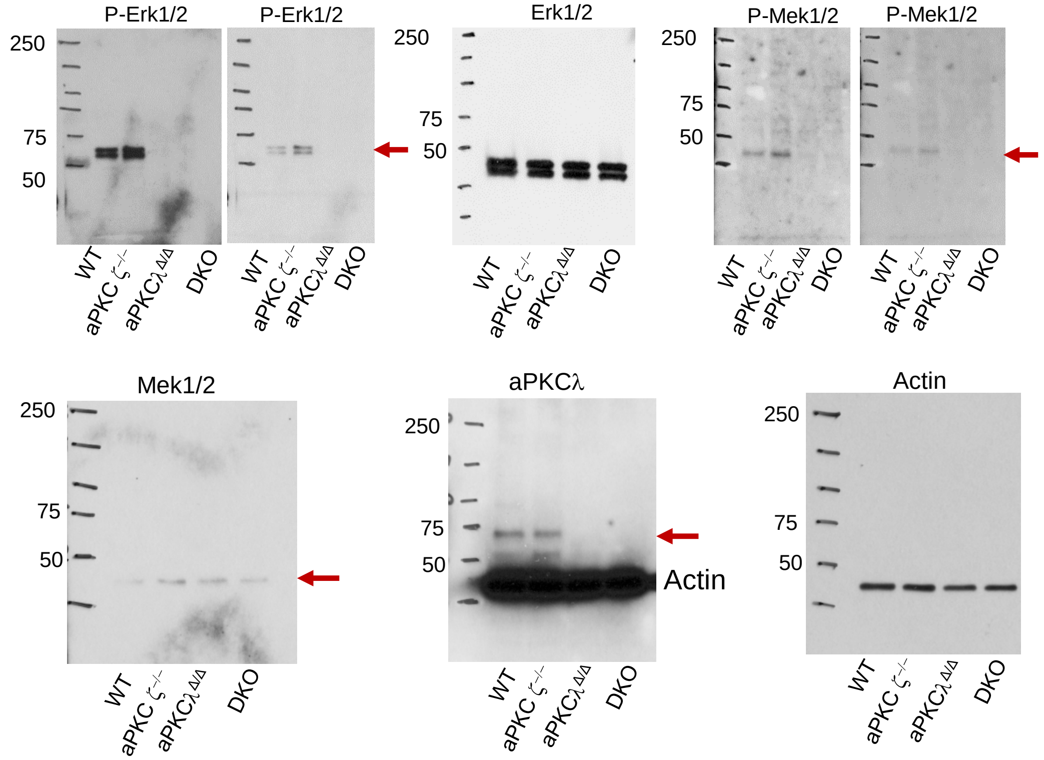

Fig3E

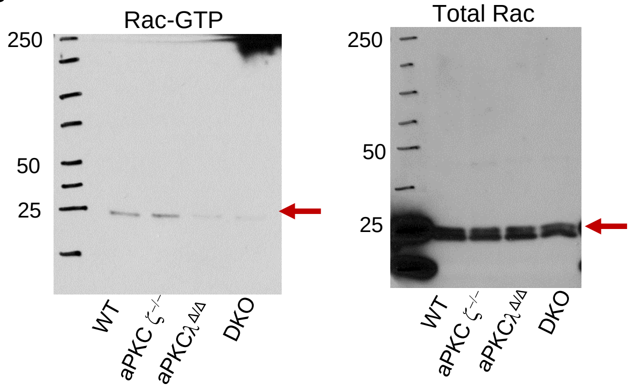

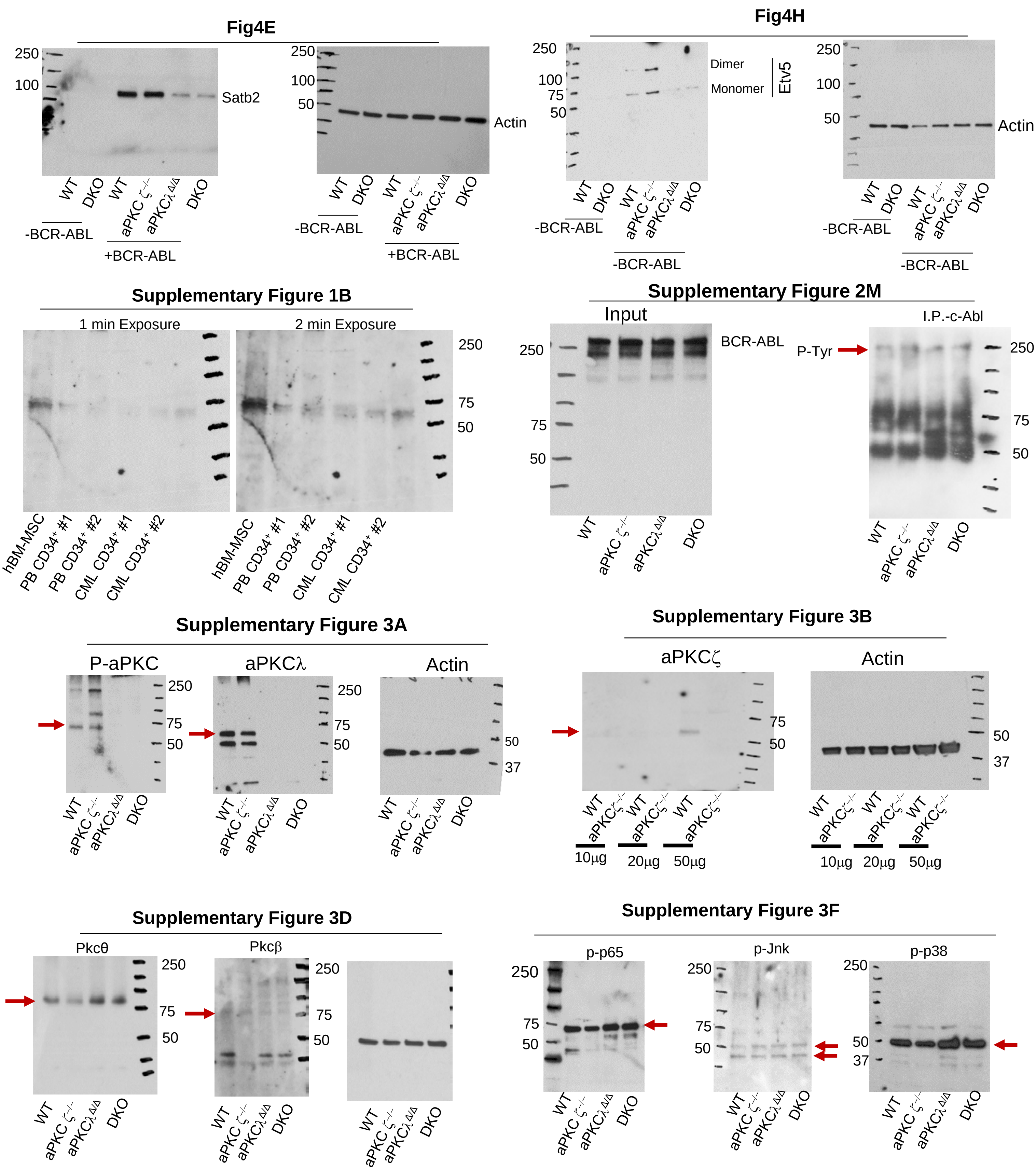
Fig4E
250
100
Satb2
WT
DKO
WT
aPKC ζ-/-
aPKCλΔ/Δ
DKO
-BCR-ABL
+BCR-ABL
250
100
50
Actin
Fig4H
250
100
75
50
Dimer
Monomer
Etv5
-BCR-ABL
Actin
Supplementary Figure 1B
1 min Exposure
2 min Exposure
250
75
50
hBM-MSC
PB CD34+ #1
PB CD34+ #2
CML CD34+ #1
CML CD34+ #2
Supplementary Figure 2M
Input
BCR-ABL
I.P.-c-Abl
P-Tyr
250
75
50
Supplementary Figure 3A
P-aPKC
aPKCλ
Actin
37
Supplementary Figure 3B
aPKCζ
Actin
10µg
20µg
50µg
Supplementary Figure 3D
Pkcθ
Pkcβ
Supplementary Figure 3F
p-p65
p-Jnk
p-p38

Supplementary Figure 3G

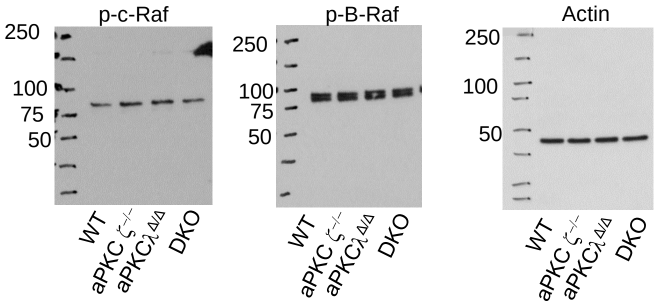

Supplementary Figure 3H

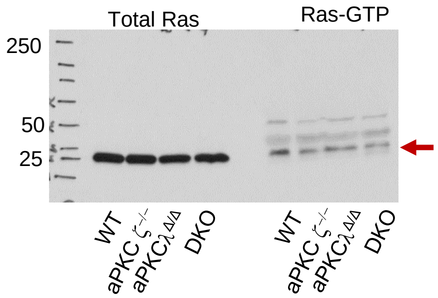

Supplementary Figure 3I

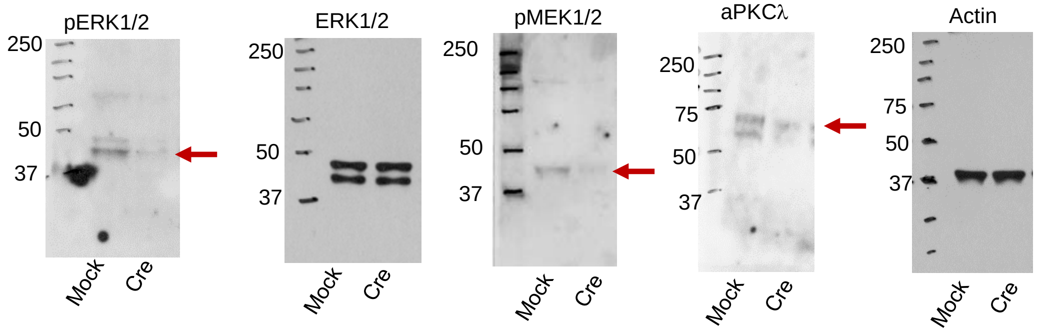

Supplementary Figure 4A

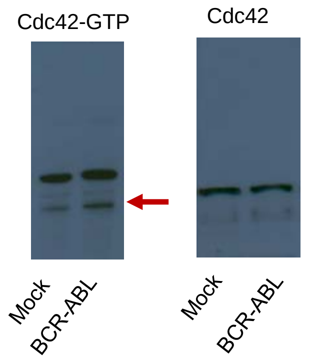

Supplementary Figure 4B

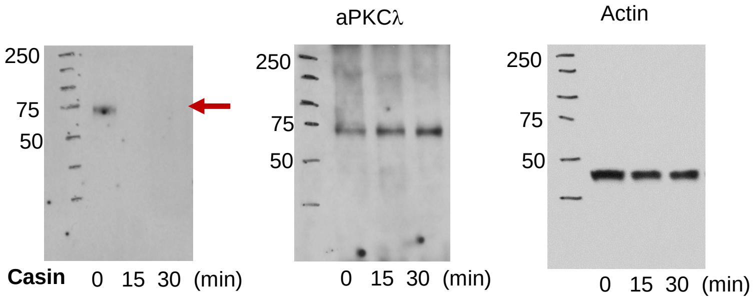

Supplementary Figure 4C

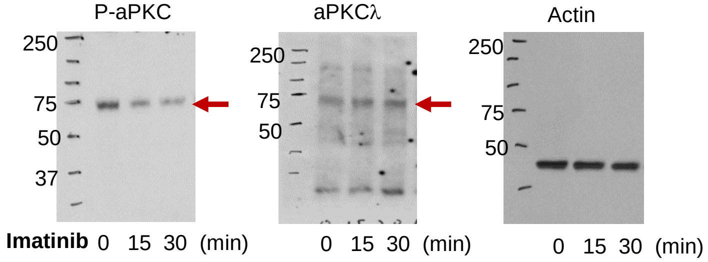

Supplementary Figure 4D

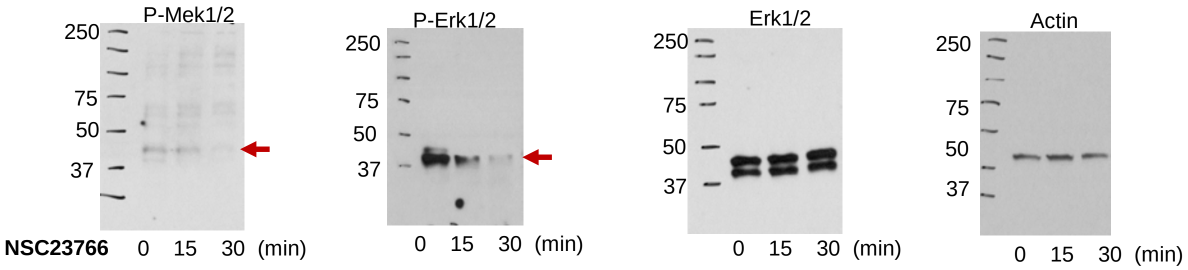

Supplementary Figure 4E

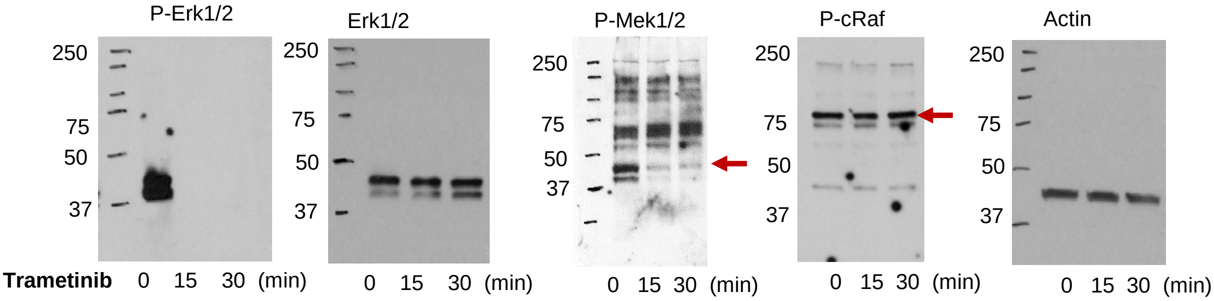

Supplementary Figure 4F

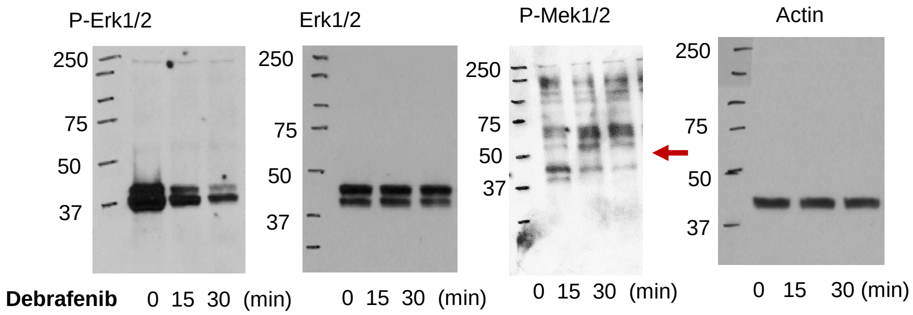

Supplementary Figure 5B

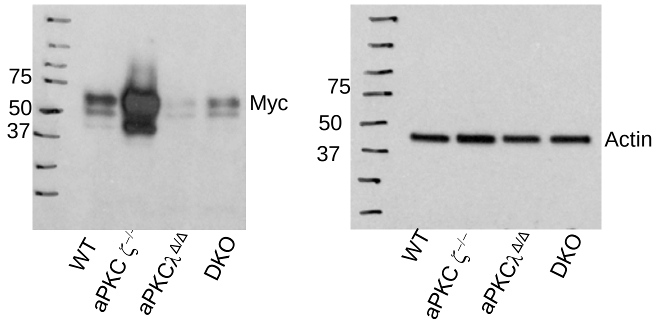

Supplementary Figure 5F

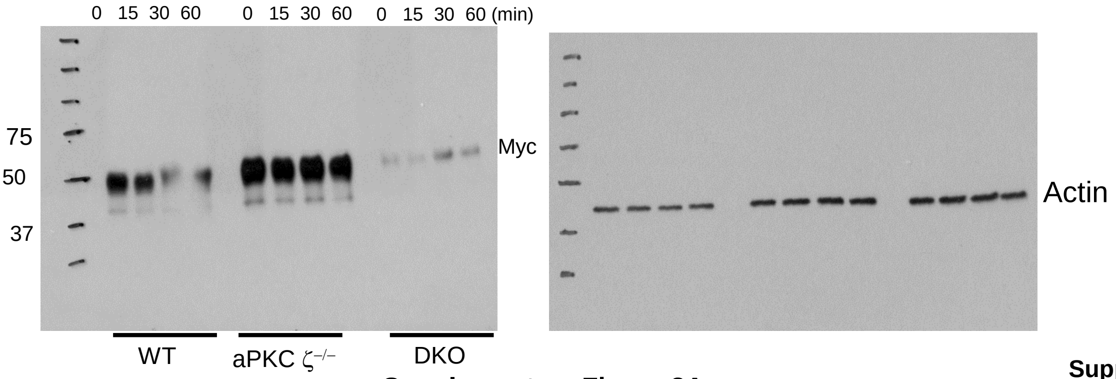

Supplementary Figure 8A

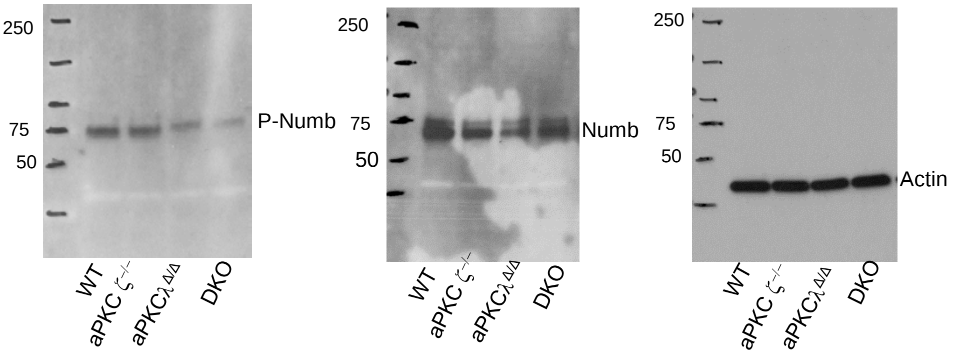

Supplementary Figure 8H

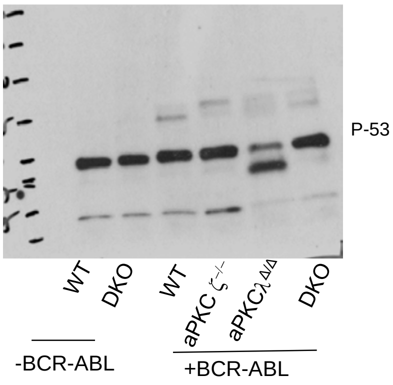

FACS gating strategy for analyses and cell sorting

Murine hematopoietic stem and progenitors flow sorting strategy

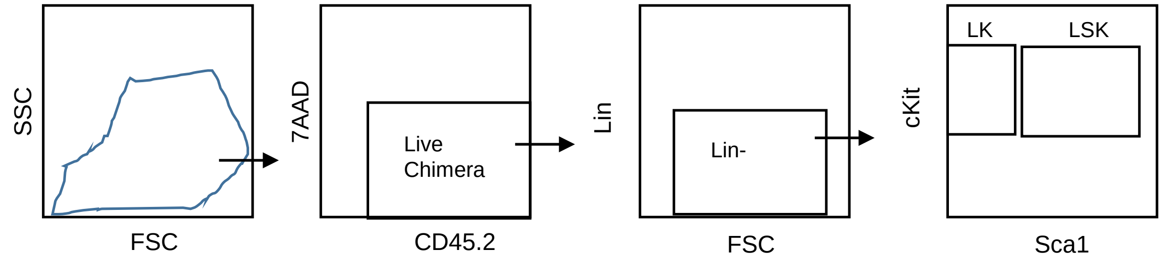

Murine B cell progenitors flow and sorting strategy

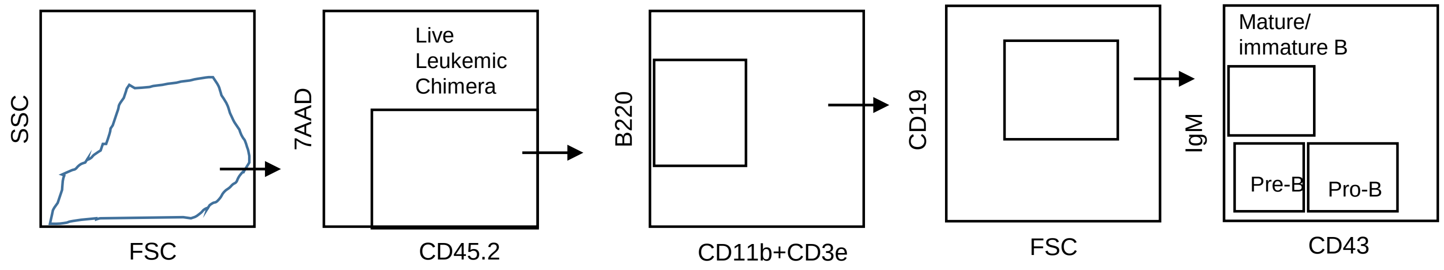

Human primary B-ALL B cell progenitors flow and sorting strategy

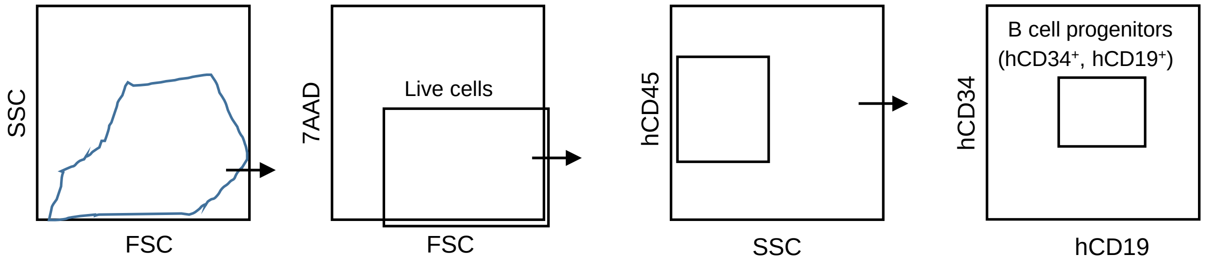

Supplement: Supplementary file 1 — Supplementary Information [file 41467_2018_7846_MOESM1_ESM.pdf]
