# Supplementary material for: The signaling axis atypical protein kinase C λ/ι-Satb2 mediates leukemic transformation of B-cell progenitors
Source: Nat Commun. 2019 Jan 4;10:46. doi: 10.1038/s41467-018-07846-y (PMC6320370; doi:10.1038/s41467-018-07846-y)
Supplement: Supplementary file 5 — Description of Additional Supplementary Files [file 41467_2018_7846_MOESM5_ESM.docx]

Supplementary Dataset Legends:

**Title:** Supplementary Dataset 1:
**Description:** The differential expression analyses of the total RNA extracted from leukemic B cell progenitors derived from WT and DKO leukemic chimeric mice (n=3 mice for each group). The raw Fast Q data were analyzed for differential expression (<0.8 to >1.2-fold, p<0.05) using AltAnalyze v.2.1.0 software developed by Cincinnati Children’s Hospital Research Foundation.

**Title:** Supplementary Dataset 2:
**Description:** Gene Ontology pathway analyses of the whole transcriptome of WT and DKO leukemic B cell progenitors (GO.DKO_vs_WT-fold1.2_rawp0.05) using AltAnalyze software v.2.1.0 software developed by Cincinnati Children’s Research Foundation
